# Why some women fail to give birth at health facilities: a qualitative study of women’s perceptions of perinatal care from rural Southern Malawi

**DOI:** 10.1186/1742-4755-10-9

**Published:** 2013-02-08

**Authors:** Lily Kumbani, Gunnar Bjune, Ellen Chirwa, Address Malata, Jon Øyvind Odland

**Affiliations:** 1 Institute of Health and Society, Department of Community Medicine, University of Oslo, Norway, P.O. Box 1130, Blindern, Oslo 0318, Norway; 2 Kamuzu College of Nursing, University of Malawi, Blantyre Campus, P.O. Box 415, Blantyre, Malawi; 3 Kamuzu College of Nursing, Lilongwe Campus, Private Bag 1, Lilongwe; 4 International health, Faculty of Health Sciences, University of Tromsø, N-9037, Tromsø, Norway

**Keywords:** Community, Health surveillance assistants in maternal and newborn care, Lay birth attendant, Perinatal period, Quality of care, Skilled birth attendant

## Abstract

**Background:**

Despite Malawi government’s policy to support women to deliver in health facilities with the assistance of skilled attendants, some women do not access this care.

**Objective:**

The study explores the reasons why women delivered at home without skilled attendance despite receiving antenatal care at a health centre and their perceptions of perinatal care.

**Methods:**

A descriptive study design with qualitative data collection and analysis methods. Data were collected through face-to-face in-depth interviews using a semi- structured interview guide that collected information on women’s perception on perinatal care. A total of 12 in- depth interviews were conducted with women that had delivered at home in the period December 2010 to March 2011. The women were asked how they perceived the care they received from health workers before, during, and after delivery. Data were manually analyzed using thematic analysis.

**Results:**

Onset of labor at night, rainy season, rapid labor, socio-cultural factors and health workers’ attitudes were related to the women delivering at home. The participants were assisted in the delivery by traditional birth attendants, relatives or neighbors. Two women delivered alone. Most women went to the health facility the same day after delivery.

**Conclusions:**

This study reveals beliefs about labor and delivery that need to be addressed through provision of appropriate perinatal information to raise community awareness. Even though, it is not easy to change cultural beliefs to convince women to use health facilities for deliveries. There is a need for further exploration of barriers that prevent women from accessing health care for better understanding and subsequently identification of optimal solutions with involvement of the communities themselves.

## Introduction

Of the estimated 2010 number of 7 · 7 million deaths worldwide in children under 5 years of age**,** 49.6% occurred in sub Saharan Africa, 33% in South Asia and less than one percent in high income countries [1]. Out of the estimated 8.8 million deaths occurring globally in the same age group in 2008, 41% occurred in neonates. Twenty-nine and 54% of these neonatal deaths were in Africa and South East Asia, respectively [2]. South-Asian and sub-Saharan African regions account for two thirds of the global burden of neonatal deaths annually [3].

Countries worldwide are striving towards achieving Millennium Development Goal 4, which deals with the reduction of neonatal deaths. High-income countries have made much progress while low-income countries still lag behind [4]. High-income countries have managed to achieve an almost universal skilled birth attendance with provision of appropriate care. Neonatal mortality rate of as low as 0.45 per 1000 live births and intrapartum stillbirth rate of 1.22 per 1000 births have been observed [4]. Countries with a neonatal mortality rate of greater than 30 per 1000 live births have about 50% skilled health worker attendance at birth [5]. Skilled birth attendant at delivery, timely emergency obstetric care, provision of immediate newborn care and postnatal care are essential in promoting neonatal health [4,6,7]. Therefore, a shift in place of delivery from home to health facilities is seen as an important strategy for improving neonatal outcomes [8].

However, it is not only availability of health facilities for deliveries that is important, but also the quality of care provided. Emphasis is being placed on the quality of care, not only on availability of services [9,10]. Lack of quality care at health facilities limits women’s access to quality care [11]. Women may deliver in health facilities, but still have poor perinatal and neonatal outcomes because of the substandard quality of care. A study in rural Tanzania showed that even at higher-level facilities where well trained health workers were supposed to be available, women experienced delays in receiving emergency obstetric care and had poor quality of care. Consequently, women experienced severe birth injuries and stillbirths [12]. When women have a choice, they will go to health facilities where they perceive better quality of care, regardless of distance [13,14]. Forty-four percent of women by-passed their nearest health facility largely because of quality of care and delivered in another health facility [15]. Women’s actual experience of care is significant and will greatly influence how women perceive quality. According to Hulton, Matthews and Stones (2000), a quality of care framework reflects both the provision of care and women’s actual experience of the care [16]. It is argued that understanding women’s experiences of care is critical as it contributes to the use of health services and perinatal outcomes [17].

Quality is not the only reason hindering pregnant women to access skilled birth attendance. Women continue to experience various problems to deliver with the help of skilled attendants. Literature suggests that women encounter sociocultural factors, perceived benefits, economic accessibility and physical accessibility as barriers to accessing skilled attendance during delivery [18]. Women in sub-Saharan Africa still face limited access to skilled delivery, especially in the rural areas [19,20].

Malawi is making efforts to reduce intrapartum related deaths as a way of achieving Millennium Development Goal 4. This is, among other health interventions, asking pregnant women to deliver in health facilities with skilled attendance. In Malawi, the perinatal mortality rate is estimated at 40 deaths per 1,000 births and the neonatal mortality rate is 31 per 1,000 live births, with 71% skilled attendance for deliveries. Skilled attendance is higher in urban areas at 84% compared to 69% in the rural areas [21]. In spite of these indicators, there is still need to increase the number of women delivering in health facilities as one way of preventing avoidable intrapartum-related deaths and neonatal deaths. This could further reduce the perinatal and neonatal mortality rates in Malawi. The argument here is that there is no justification for deaths due to intrapartum-related complications.

Despite the policy of Malawi government that women should deliver in health facilities with skilled attendants, it is not guaranteed that women will adhere and deliver in health facilities. Perception of quality is important in influencing the place of delivery, although it may not be the most important reason why women fail to access health facilities. Information on the views of women on perinatal care is limited in Malawi. The objective of this study was to explore the reasons why women delivered at home without skilled attendance despite receiving antenatal care at a health centre and their perceptions of perinatal care.

## Methods

### Study design

The study was a descriptive qualitative study that used a semi-structured interview guide. Face-to-face in-depth interviews were conducted. Participants who delivered outside a health facility between December 2010 and March 2011 were asked to describe their perception of perinatal care. The women were asked how they perceived the care they received during antepartum, intrapartum and postpartum. They were also asked about the information they received during provision of care. The interviews were conducted in the local language, Chichewa, using semi structured interview guide. During the interviews, follow-up questions using probes were asked in order to acquire a deeper understanding when an explanation was unclear. The interviews lasted on average, 45 minutes. All interviews were recorded, translated and transcribed verbatim in English.

### Setting

The study was conducted in the Southern region of Malawi in the Chiradzulu district. Out of the 10 health centers under Chiradzulu district hospital, the catchment area of Namadzi health centre was selected for this study because many women from that area delivered at home with traditional birth attendants (TBAs) as revealed by hospital records of the period July 2008 to June 2009. The records showed that the Namadzi catchment area had the third largest number of expected pregnancies of 1380, but low skilled deliveries of 853 representing 62%. This was compared to the first two health centers that had 92% and 97% of skilled delivery respectively. Data were collected from mothers who received antenatal care at Namadzi health centre, but delivered in the community without skilled attendants.

### Participants’ recruitment and data collection

Mothers who attended antenatal care at Namadzi health centre but delivered outside the health facility, either with TBAs or at home in the course of the study were selected using purposive sampling. All women who were approached and asked to participate in the study accepted. The age range covered the youngest primipara and the oldest delivering mothers in villages. This was done through reviewing postpartum records of mothers who came to the health centre after delivery as well as asking these mothers if they knew anybody else who had delivered in the community. The health surveillance assistants reviewed the records, and then went to the villages to trace the mothers. Health Surveillance Assistants (HSAs) were used in the villages to help trace the names of mothers that delivered during the study period at home and with TBAs from community members. The register of the Village Headmen did not have this information. The same HSAs traced the mothers in their homes. The first author provided information to and recruited potential participants that met the defined criteria. The HSAs organized with local leaders such as Village Headmen, headmasters and church leaders to provide a suitable place where the interviews were conducted. The first author conducted all the interviews. The interviews were done in a church, school building or out in the open air. The researcher ascertained that the place used for the interviews was appropriate to maintain privacy and comfort. A total of 12 mothers had been interviewed in the community when saturation of data was reached. There was no new information that was coming. Instead, there was repetition and confirmation of already collected data [22].

The 12 mothers were from eight different villages namely Masuku, Walala, Kachere, Ulaya, Chelewani, Matola, Mng’omba and Lidala. Masuku village, 3.5 km from Namadzi health centre, is the closest one to the health centre. It is mostly flat land and it is close to the main road. Chelewani is a hilly area which is 4 km from the health centre. Lidala is flat with rivers and it is 5 km from the health centre. Ulaya is also 5 km from the health centre and it is located along the main road and it is flat. Kachere is located 6 km from the health centre and its terrain is hilly. Walala is 6 km from the health centre, along the main road. Matola is a flat area 7 km from the health centre. The furthest is Mng’omba, with hilly land and 10 km from the health centre. Its road is in bad condition with plenty of potholes. There is no public transportation available within the villages and only dry weather roads. The people have to walk to the main road in order to access transport such as minibuses to the health facility.

### Data analysis

The Atlas. ti version 6.2 computer software program was used to code the transcripts and store the data. The Statistical Package for Social sciences (SPSS) version 18.0 was used for data entry and descriptive analysis of the participants. Analysis focused on participants’ perception of the care they received during antepartum, intrapartum and postpartum. Specifically, attention was on what they liked about the care they received and what problems or constraints they faced. All recorded interviews were transcribed verbatim in full. The analysis focused on developing coding categories where narrative information was organized according to emerging themes using thematic analysis [23]. Coding of the data was done without fitting it into a preexisting coding frame. The data were read several times to identify themes that were related to quality of care.

### Ethical consideration

Approval for the study was granted by the Norway Regional Committee for Medical Research Ethics as well as the College of Medicine Research and Ethics Committee (COMREC) in Malawi. A written permission to conduct the study was obtained from the District Health Officer (DHO) of Chiradzulu District Hospital.

Participants who agreed to participate in the study gave written informed consent or a thumbprint for those who were illiterate. Participation in the study was voluntary and participants were assured that anonymity would be observed at all times. Confidentiality of participants was maintained by using numbers on both the recorded interviews and transcripts.

### Limitations

The study might suffer a bias because the participants were women who delivered at home within three months of the study. Consequently, it excluded the rest of the women who delivered at home who might have provided another view. Data were collected during the rainy season, possibly affecting movement of mothers to the health facility for delivery. It was not possible to go back to the community for member checking because of resource constraints. However, the findings are consistent with findings from other studies reviewed. The first author who collected the data is a health professional, which may have influenced some of the participants’ responses.

## Results

### Demographic data

Twelve in-depth interviews were conducted. All participants were married. The majority (n = 9) of the participants had primary education. The age range was 20 to 32 years, with an average age of 24.5 years. None of the women below 19 years and above 33 years of age delivered their babies at home. Parity of the participants ranged from one to seven and the majority (n = 9) of the participants had given birth between two to four times. A few (n = 4) participants were assisted to deliver by traditional birth attendants (Table 1). A few of the participants (n = 5) came from one village (Masuku), with the rest spread one per village.

**Table 1:** Demographic characteristics of participants (n = 12)

**Characteristic**	**Number**
**Education level**	
No education	1
Primary	9
Secondary	2
**Age group**	
20-24	6
25-29	4
30-34	2
**Parity**	
1	1
2-4	9
5 and above	2
**Person who assisted the birth**	
Traditional birth attendant	4
Relative	3
Neighbor	2
A passerby (stranger)	1
None (self delivery)	2

### Antenatal care

#### Perception of good care

The participants perceived that the care they received during antenatal was largely good because they were given advice on what to take when going for delivery. They received medicines as prophylaxis against anemia, malaria as well as actual treatment of malaria when they were ill. For some participants, the prophylaxis they received reduced malaria attacks and dizziness. Additionally, they received mosquito nets that protected them from malaria. The participants’ narrations indicated that because of the antenatal care they received, they did not experience any problems. The delivery process went well and they had healthy babies. Participants rated care as being good when they were warmly received and not treated harshly at the clinic as explained by participant T:

“When others went (antenatal clinic) they were treated harshly but when we went we were not in any way. We were warmly welcomed”.

Participant Q shared on abdominal palpation as follows:

“They were not rough as they are to some when examining them. They examine roughly, being rough squeezing very hard (on the abdomen)”.

#### Perception of poor care

Participants perceived poor care as being shouted at and delay in receiving care. Participants expected health workers to be friendly in providing care and not to be rude or shout at them. Some health providers were said to be rude and participants wanted them to change and not shout unnecessarily. For instance, when they go to measure their weight, care providers said that they were wasting their time or that they were not sitting properly as directed when waiting for assessments. A participant reported that she expressed concern about the shouting as she was worried how they would be treated during labor if they came to the same facility. She stated that another health worker also queried the shouting at the clients. The health worker’s response to the participant was deplorable. The participant shared the experience as follows:

*“I asked if you are doing this when labor started and I come. How is it going to be? It will be the same, shouting at us? That day you will even beat us then? She said, yes if a person is troublesome, we beat her up. We are very annoyed with some who exaggerate and cry when giving birth”.* Participant P

Another concern was delay in the provision of care. Participants did not like being kept waiting at the antenatal clinic when it was opened late. They also disliked when they were not attended to when they went to the health facility. A participant who was admitted after presenting with malaria in pregnancy was not happy with how she was managed. She went to the health facility but was not cared for as had been promised. They gave her medicine the next day in the morning. She felt they did not show her kindness and shared as follows:

*“It was 12 midday. It is late and we are tired. You will be seen later after lunch when our colleagues come for the next shift. They took the book (health passport which has antenatal information) and gave me a bed. I ended up sleeping until in the morning. This hurt me because they told us that when we have malaria, we should go to the hospital quickly. Yet here I was I slept without receiving medicine until the next morning”.* Participant X

#### Labor and delivery care

All participants who delivered in the community had intended to deliver at the health centre. Some even admitted that despite being informed during pregnancy to go to the health centre in advance and wait there for delivery, they delivered at home. The participants gave various reasons why they failed to deliver at the health facility. Participants reported that when a woman is in labor and does not even have a bicycle, she can not walk to the hospital. For this reason, most women deliver on the way to the hospital. A woman in labor will walk for a short distance and feel she can not walk any longer, then just goes to the TBA. A participant took blame for delivering outside a health facility and explained as follows:

*“For an individual to receive care is up to yourself. When you know now you are in labor, go quickly to the hospital. At the hospital they would care for you because they see you are between life and death. They would see how to help at that time because you have submitted yourself. To stay away is what happened to me. I delivered in a garden and l did not reach the hospital”*. Participant X

#### Reasons for place of delivery

A few participants reported that labor started at night. Some of them started off for the health facility but delivered in transit, while others stayed and delivered in their homes. Participant P narrated as follows:

“As we were walking on the way I could not proceed because I failed to walk. Then they went and called the TBA. They told her that we were on our way to the clinic and I was on the road side. As soon as she arrived she delivered the baby”.

A few participants reported that they delivered on the way to the hospital because labor was quick. One participant delivered alone at home because of the rains. She could not walk in the rains. She explains as follows:

*“I was ready but it started raining. People were informed but when they came they found the baby was delivered”.* Participant N

Participants went to a TBA because they had difficulties in walking and TBA was closer than a health facility. They could not walk because one had a swollen leg, and the others had rapid progress of labor. One of the participants said she failed to walk because labor had advanced and had to be carried to the TBA at around 4 in morning. She delivered at night around 9 o’clock. She reported that when she arrived at the TBA, she was told there was still time before she could deliver because the cervix had not yet dilated. She wondered if she was bewitched. Participant O shared that labor progressed quickly. She said the following:

“I wanted to deliver at Namadzi but it was during the rainy season. When labor started, with the rains and the speed with which labor progressed and it affected my legs. Furthermore, the baby quickly descended and was soon delivered”.

The participants expressed lack of control over the actual place where they delivered. The participants narrated that labor started unexpectedly when they were sleeping and they had no men to escort them at night. Some participants started off, going to the hospital but saw they could not continue and ended up delivering by the roadside or in a maize garden. Others accepted fate about whatever might happen when they delivered at home or in transit. All the participants delivered live babies. Participant Z shared as follows:

“It was around 11at night. I had to get ready as well as wake up other people. I woke up my mother then she had to wake up others for us to be a group as it was at night. Then we started off and just walked a short distance when I could not walk anymore. They asked, ‘you will not manage to walk?’ and I said ‘no’. ‘What are we to do they asked?’ One suggested that they carry me to the bus stop but I refused that it would not work. It was better for me to deliver there because my grandmother knew and she would assist me. They said, ‘no we should not break the hospital rules’. I asked, ‘what are we going to do? Let us just do it and hope we will manage. We will go to the hospital later’. They agreed. I just lay down on the roadside and delivered”.

#### Confidentiality by birth attendants

Participants who were delivered by TBAs stated that TBAs maintained confidentiality, as they did not say anything about delivery issues. TBAs were said to be very secretive about what happened during delivery. Participant P said:

“By not saying anything, after delivery you do not hear her telling other people about you. That one this and that, it ends with delivery and you never hear anything”.

Participant T, who was assisted to deliver by her neighbor in transit, shared that confidentiality was also maintained.

“When I delivered on the road we went to the hospital and no one knew I had delivered on the way to the hospital. Up to this day, people do not know I delivered on the way to hospital”.

This was different for participant S who was assisted to deliver by a neighbor as well, but confidentiality was not maintained. Her neighbor went about telling people that she delivered the baby and that if it were not for the neighbor she would not have managed to deliver.

#### Conduct and attitudes of birth attendants

Participants who delivered at TBAs reported they were well received when they went for delivery. Participants compared care they received at traditional birth attendants with that at health facilities. Participants expressed that care at the TBA was not adequate and that they preferred to deliver at a health facility. This was because TBAs just deliver but they do not know about problems and what to do. At a health facility, one receives adequate care. If they see you have not delivered well, they give you medicine or refer you to a big hospital. This is true because TBAs are not be able to manage complications when they occur. Participants were not happy when they waited for the TBA on arrival. They were also not happy that they were left alone during labor because they were in pain.

Participant O explained:

“I don’t know what she was doing at her house. You know how proud doctors are! Just like in hospital when you arrive they do not attend to you immediately. She (the TBA) was a bit cruel because during that time one is in pain. It is a difficult time”.

A participant who was left alone during labor and the TBA came to conduct the delivery said the following:

*“When she examined me she said the cervix is not dilated she told me to lie down. When it’s close you will know as you are a big person. Then she would leave me; go wash her plates. When I was having pains I just endured. When I felt that the baby was about to be delivered, that is when I called the TBA. When she came she wore her gloves, two minutes later the baby was delivered”.* Participant E

None of the participants talked about technical aspects of care during labor and delivery. However, they focused on how they should have been received and treated at health facilities. It was only mentioned that one received adequate care because resources were available. When a client is on the bed, she is given a bedpan and kidney dish and told what to use for what. Therefore, one is able to do things easily regardless of the stress of labor. If you want to vomit, you use the kidney dish.

Participants wanted health workers to be available and close by to avoid self deliveries, without their assistance. The health workers’ role was mainly viewed as conducting the delivery. One of the participants, a grandmultipara, delivered only the first baby in a health facility. She delivered the next babies at a TBA because she said the TBAs were working at that time. She did not know that care provided during labor was more than assistance during delivery. She said that:

*“When you are in labor you wait for delivery of the baby. I do not see anything else that could happen”.* Participant A

#### Postnatal care

Nearly all of the participants went to the health facility after delivery. Participants that delivered in transit proceeded immediately to the health facility while the ones at home waited for morning if they delivered at night. One participant went to the health facility a week after delivery. The majority of the participants and their babies were assessed at the health centre. The mothers who were not examined were asked if they had any problems. When they reported they were alright they were told they would not be assessed. However, cord care was performed and majority of the babies had their birth weight checked.

### Reception at health facility

A few participants reported they were well received at the health facility. The reasons were that they were assessed without any problems, informed of the findings and not shouted at. Cord care was given, babies were weighed and a bed with linen where they slept was provided. Participant S explained as follows:

“I was well cared for. There are some doctors who when a patient arrives in hospital, will not give her the care befitting a human being but will just look at her unconcerned. But when I arrived, a doctor immediately told me that he needed to examine me to see if I was fine”.

A few participants stated they were shouted at when they arrived at the health centre before being given care. This was because they had delivered at home and not in the health facility as advised by health workers. Participants stated that they were told to go and wait at the facility at term gestation. It was also said that there is a village bylaw that requires all pregnant women to deliver in health facilities. Participants gave reasons for failure to wait for delivery at health facility. Some of the reasons were fear of people who could bewitch a pregnant women not to deliver on time even after the onset of labor, responsibilities such as looking after an elderly mother who was at home alone that made it difficult to leave and lack of a guardian. Relatives were reluctant to be guardians at the health facility as they were busy in their gardens. One participant mentioned she was just lazy. Participant N narrated as follows:

It was crop growing season, people are reluctant to help and be with you at the hospital. They say “I have lots of weeds in my garden. You may go there and stay up to a month. Others start complaining ‘she made me leave my work just to sit here, my crops are suffering’…When you say let us go they will respond that you should wait until it is time. Instead of going there to wait for delivery and stay up to a month. Others have gone there and waited up to two months before delivery. Then you feel like you will trouble people”.

#### Attitudes of health workers

A few participants expressed that the health workers were rude. Participants explained that others after they deliver in transit, they are told that they will not be assessed at the health facility and should go to Chiradzulu district hospital if they wished. A participant was told she should deliver the subsequent pregnancy at Chiradzulu district hospital. Others accepted being shouted at because they did not follow advice. They even said that they were well received. A participant who delivered at a TBA and had retained placenta shared as follows:

*“I was well received. I was not shouted at much. …… At the hospital they said they had told me to go and wait for hospital delivery but l chose not to listen”*. Participant O

Participant X who delivered at 4:04 in the morning used a minibus to go to the health facility. They arrived just before 5 in the morning on the same day. She related what happened as:

“The nurse we found told us you are late, where were you all this time that you are only coming now? The nurse went out and left, and we wondered where we were going to stay. I was informed to go to the ward by another health worker who came not long after the other one who had left. Then I went to the bed. I had mud all over as you know how it is during the rainy season. I went to the bed and I thought it is not right that I get onto the bed with all this mud. He came and told to get on the bed and I did”.

#### Provision of care

Participants’ stay at the health facility varied from leaving immediately after assessment to spending a day. Some participants who came in the morning were discharged the same day in the afternoon, while others were given a choice to stay or go back home. A participant was told to go home because the ward was full, she had delivered well, and the baby was fine. The participant explained as follows:

*“I felt I was given good care, even though they told me to go home. I saw that there was no problem. If I had a problem, I would have been admitted and they would have assisted me”.* Participant A

Although participants stayed at the health facility after delivery, some of them reported that postnatal assessments were not done on them or the babies. Health workers only weighed the babies and informed the mothers they would not be examined because they were fine. A participant who was kept at the facility over night reported that she and the baby were not examined either on admission or on discharge. The health worker only clamped the baby’s cord. The participant wanted health workers to examine the baby and herself. However, her narrations showed that some form of assessment was performed on the baby. She shared as follows:

*“At the hospital they took him and asked us to warm some water. They took the water with small piece of cloth wiped him. Then they did the cord, wrapped him and placed him on the bed”.* Participant P

When mothers went to the health facility some of the babies were given immunizations on the same day, while others were given during postnatal check up visits. All of the participants were told about taking their babies at one week for postnatal check up. Health information about breastfeeding and advise to keep the baby warm was given to the majority of the participants, followed by advise to keep the baby warm. Other topics that were mentioned were cord care and family planning. Only a minority of the participants were told about danger signs of the baby. Some participants mentioned strange practices of cord care that they were told at the facility such as application of vaseline on the cord. Participant S explained her postnatal check up as follows:

“When I went there they gave him an injection on the arm (BCG) and oral drops (Polio o dose). Then I went to labor ward where he was examined and they documented in his book (health passport)”.

Another participant reported that she was turned back when she went for postnatal check up and was given another day to go back. She however did not go back; instead she went to an under-five clinic.

*“I told them I had come for (postnatal) check up. Then we were sent back and told to come another day, Thursday. From there I went to the under-five clinic where they said the baby was not old enough to start under five clinic. They took his book and wrote in it and told me to take him inside. When I went in, they gave the baby droplets in the mouth (polio 0 dose) and they told me to come back after two weeks”.* Participant P

#### Previous labor and delivery experiences in health facilities

Participants narrated different situations of their previous experiences of labor and delivery care in health facilities. Participants explained how they met rude health workers that spoke and treated them harshly during labor and delivery. Some of the participants were left and delivered alone, and stopped from holding the headboard of the bed to support oneself to push effectively and deliver quickly. A multipara who delivered the previous baby alone in a health facility said she was ill-treated and concluded the health facility is good but at times it is not. She shared her experience as follows:

*“The third pregnancy when I went to the hospital, the doctor that I found was harsh. This made me say the hospital is good but at times it is not. I was with my mother and went to the ward where people wait. The doctor was called to come and she came. She simply stood at the door and just said it was not yet time, wait. My mother told her that I had started some time back but as she said that it was not yet time she could go. She left and went back home. I delivered alone since I have delivered before. I just persevered until the baby was delivered”.* Participant U

Participants stated they wanted to be treated like human beings, with respect and not shouted at when they go to health facilities. When women go to a health facility they are in pain and health workers should not shout or ignore them. Health workers should attend the women quickly when they arrive at health facilities. A participant, a multipara related labor and delivery process to being mentally disturbed when you can say anything to a health worker, even use swear words without retribution. She shared as follows:

*“This time is as if you are mentally disturbed you can say anything to the doctor. You can even use swear words but most doctors will disregard what you say. But you can find some doctors who will even slap you – ‘how can you say that to me?’ When you deliver you think what was it I was I doing. During that time you disregard stuff and when they do not things do not go well…. a lot is said there (at the health) that is why many go to the TBAs”.* Participant Z

## Discussion

A way of reducing perinatal morbidity and mortality is to get more pregnant women to deliver with the assistance of skilled attendants. Understanding perceived barriers that prevent pregnant women from delivering in health facilities is a step towards focusing on how to help pregnant women to reduce or eliminate these factors.

Of interest on the demographic characteristics of the participants is that most women who delivered at home were those that were previously classified as low risk. This is in terms of their age and parity. Risk assessment was done antenatally and clients were explicitly recommended where to deliver [18]. The low risk category of women was even allowed to deliver with trained TBAs before the concept of skilled birth attendants for all became the focus. It may be the same reasoning behind the finding that when the women knew during antenatal care that they had no problems or complications with the pregnancy, they felt it was safe to deliver outside a health facility. A study in rural Tanzania demonstrated that women with ‘normal’ pregnancies expected to deliver at home with no problems. Health workers reported antenatal care provided the women with reassurance that their pregnancies are normal [24,25].

Evidently there is need to increase women’s awareness on the necessity of skilled birth attendants during delivery to ensure that women deliver in health facilities and neonates are given appropriate care. Women need to understand through targeted health information messages that complications may occur without warning anytime during labor and delivery; and even after completely ‘normal’ pregnancies.

It is not possible to say why fewer than half of the participants who delivered outside the health facility were from Masuku village. This is closest to the health centre out of the rest of the villages. Its terrain does not make this village more difficult to access the main road than that of the rest of the villages. However, more than half of participants from this village talked about the issue of self-delivery in health facilities while some had beliefs that witchcraft would make pregnant woman delay in giving birth. An earlier study done in Malawi also found that more than half of the participants believed it was possible to be bewitched during labor. This belief did not change post intervention [26]. Maimbolwa, Yamba, Diwan, & Ransjo-Arvidson (2003) showed that a pregnant woman should not reveal labor had began to avoid complications during labor and delivery caused by evil spirits and witches in Zambia [27]. The women may prefer to wait and go to a health facility until labor is well established, so that it cannot to be stopped by witchcraft. In this way, they delay going to the health facility or they do not comply at all. Choudhury & Ahmed (2011) revealed that in rural Bangladesh, traditional beliefs delayed care-seeking in health facilities [28]. This belief about witchcraft needs to be addressed for communities not to relate labor and delivery process to witchcraft. Of course, a simpler mechanism might be that those who are closest to the clinic take the risk of waiting until the last minute, and then most often fail to reach there. Provision of appropriate perinatal information by health workers is necessary to raise community awareness.

Additionally, health workers must strive to avoid self-deliveries when women go to deliver in health facilities. Self-deliveries deter women using health facilities with subsequent pregnancies [29,30]. Use of a companion to support a woman during labor is associated with a safe and satisfying birth experience [31]. Due to shortage of health workers they are not in a position to meet all the needs of a woman in labor. Guidelines in Malawi for the provision of care stipulate that a support person be present during labour and delivery [32,33]. Using a doula to be with the women provides a one to one support to the women as they do not take care of anyone else [34,35]. A study in Malawi has shown that supportive companion during childbirth is highly acceptable among mothers, health professionals, and the community [36]. Implementation of this strategy may address women’s concern of being left alone during labor in health facilities in Malawi.

Women’s views on quality focused on how they were received at antenatal clinic and when they went to the health facility after delivery. Participants stated care was good at antenatal clinic and in labor ward based on good reception and what was done. The women did not complain about the technical quality of care. This is similar to findings of women who delivered at a district hospital in the same district [37]. The authors have shown that women are often not critical to the care they receive. The women did not know the quality of care to expect because they were not well informed, ending with higher risk for delivery problems. The major concern for the women in this study was poor staff attitudes. Health workers shouted at the women and even threatened to beat the women if they would be troublesome when they went for delivery. Other studies confirm that poor attitudes from health workers, who were rude and abused women, discouraged the women from delivering in health facilities [19,24,30,38,39]. This may provide an explanation why some of the women did not deliver at the facility. It was perhaps fear of abuse from health workers though they said otherwise. Humane aspect of care matters greatly in provision of care to women during labor and delivery [40,41]. A laboring woman is vulnerable and in pain thus needs understanding. Positive attitudes and empathy of health workers was related to delivery in health facilities [39,40,42].

There is an urgent need to address the poor attitudes of health workers for them to provide appropriate professional midwifery care to women. Frequent supportive supervision of health workers at the health facility is necessary to resolve problems they experience that negatively impact on provision of care. This is important, as health centers are understaffed; the numbers are not adequate against the workload affecting provision of care [43,44]. Management support and fairness in managerial practices contribute to improving health workers’ motivation and performance [45-47] resulting in provision of optimal care to clients.

Delivery at a TBA was associated with inadequate care because the TBA is not in a position to identify and manage complications. Despite knowing this, women still ended up delivering at TBAs and home. Probably the women felt they would have normal deliveries therefore; there was no need to go to a health facility. Research findings in Indonesia revealed that some community members preferred TBAs and home deliveries despite presence of a village midwife in the village. Specifically, services of skilled birth attendants were perceived important only during complications [48,49]. Delivery process was viewed as easy by Bolivian women therefore there was no need for them to go to the hospital [50]. Comparable, women’s experiences of uncomplicated home birth made these women think that delivery in a health facility was not necessary [30].

Furthermore, the women in this study could not go to wait at the health facility because they had no guardians to take care of their needs whilst there and other responsibilities at home. Therefore, the women may have preferred to go in established labor, deliver, then go back home. In view of this, the solution by authorities that all pregnant women wait at health facilities for delivery is not feasible for these women. This is regardless of various factors; time of the day when labor starts, lack of people to escort a laboring woman at night; and rapid labor and rainy season that hindered the women’s access to health facility. The TBAs are then seen as a ready source of help when these problems that are beyond their control hinder access to health facilities [19,24]. Habit also played a significant role, like a grandmultipara who delivered only a first child in a health facility and subsequently delivered at TBAs with no problems. Therefore, mention of rapid labor or labor starting at night could be an alibi for home deliveries [51]. Health workers should be aware of these problems and discuss them with the women during antenatal care and communities when they discuss birth preparedness. There is also need for a more thorough exploration of these barriers that prevent women from accessing health care for better understanding; and, subsequently work with the communities to identify best solutions that will be ideal for them.

As women continue to deliver with TBAs and at home, there is need to improve initial care given to babies by using health surveillance assistants that are already trained in community based maternal newborn care package (CBMNC) or train them in places where they are not available [52]. This is an important strategy as, currently, Malawi is still far from providing skilled birth attendants at home.

Distance has been recognized as a major barrier to delivery in health facilities [18,20,53,54] but distance was not found to be an issue in this study.

Assessments of some mothers and babies or delayed assessments when they went to the health facility after the mothers had delivered was inappropriate. Babies are vulnerable especially during the first 24 hours after delivery [55] and thorough assessment during this time is consequently not only necessary, but crucial. The babies merely had cord care and weighing, with few babies that did not have their birth weight checked. This may discourage other mothers who deliver outside health facilities to go to health facilities as required for review and appropriate management. Guidelines stipulate that women who deliver outside of a health facility should go to a health facility for a postnatal check-up within 48 hours of giving birth [56]. Only 21% of babies born at home in Malawi had a postnatal check after delivery within 42 days and barely 18% within 48 hours of delivery. It was even less for Chiradzulu district at 6% and 10% within 24 and 48 hours of delivery, respectively [57]. Essential neonatal care practices such as keeping the neonate warm, cord care and initiation of exclusive breastfeeding may not be done or wrongly done outside the health facility. Skilled attendants must provide care to a neonate immediately after delivery [58]. It is therefore imperative that all the babies are assessed and given suitable care when they arrive at a health facility. A baby was wiped with warm water, cord care done then wrapped. This indicates some sort of assessment was done on the baby but the mother felt that the baby was not examined. This is probably because the mother was not told about the examination. Consequently, there is need for health workers to improve in the way they communicate with clients.

Appropriate information on baby care and danger signs of the baby should be provided to all mothers before discharge. This will enable mothers to properly take care of their babies and seek care promptly when they encounter any problems [59,60].

## Conclusion

It is important for health workers to provide comprehensive client friendly care to all women when they go to health facilities to promote utilization. This will guarantee provision of essential neonatal care to babies at birth. Onset of labor at night, short labor, rainy season, and health workers attitudes were significant barriers for the women to deliver in a health facility when labor started. Nevertheless, waiting at the facility was not a viable option for the women. Socio-cultural factors were related to women delivering at home. This study reveals beliefs about labor and delivery that need to be addressed through provision of appropriate perinatal information to raise community awareness. Even though it takes time to change cultural beliefs and women’s use of health facilities is not that simple, it can be done. There is need for further exploration of barriers that prevent women from accessing health care for better understanding and subsequently identification of optimal solutions with involvement of the communities themselves.

Definition of terms

Community:

people living in an area outside Chiradzulu district headquarters and designated town centre, mostly involved in subsistence farming as the major economy activity.

Health surveillance assistants in maternal newborn care:

health surveillance assistants trained in community based maternal newborn care package (CBMNC) model to do home visits and community mobilization activities to enhance preventive care and promote use of facility care during perinatal period.

Perinatal period:

from 28 completed weeks of gestation to 7 completed days after birth.

Quality of care:

focuses on both the provision of care as well as how clients perceive the care.

Skilled birth attendant:

a skilled attendant is an accredited health professional such as a midwife, doctor or nurse - who has been educated and trained to proficiency in the skills needed to manage normal (uncomplicated) pregnancies, childbirth and the immediate postnatal period, and in the identification, management and referral of complications in women and newborns.

Lay birth attendant:

a person without any training in midwifery skills who assisted a mother to give birth. It was ones mother, grandmother, a neighbor or a traditional birth attendant (TBA).

## Competing interests

The authors declare that they have no competing interests.

## Authors’ contributions

LCK conceptualized the study, collected, analyzed data and drafted the manuscript. JØO is the Principal supervisor of the study. GB, EM, AM and JØO have critically reviewed drafts, edited and provided important intellectual content. All authors read and approved the final manuscript.
